# Establishing the Ideal Conditions to Create an Airway Fire Using a Porcine Airway Model

**DOI:** 10.1002/oto2.36

**Published:** 2023-02-23

**Authors:** Andrew M. Bysice, Tyler Oswald, Luis E. Mendoza Vasquez, Francisco Laxague, M. Elise Graham, Ruediger Noppens, Kevin Fung

**Affiliations:** ^1^ Department of Otolaryngology–Head and Neck Surgery, Schulich School of Medicine & Dentistry, London Health Sciences Centre Western University London Ontario Canada; ^2^ Department of Anaesthesia and Perioperative, Schulich School of Medicine & Dentistry, London Health Sciences Centre Western University London Ontario Canada; ^3^ Department of Head and Neck Surgery Hospital Aleman of Buenos Aires Buenos Aires Argentina

**Keywords:** airway fire, bipolar, monopolar, porcine model, surgical fire, tracheostomy

## Abstract

**Objective:**

Airway fires are a rare but devastating complication of airway surgery. Although protocols for managing airway fires have been discussed, the ideal conditions for igniting airway fires remain unclear. This study examined the oxygen level required to ignite a fire during a tracheostomy.

**Study Design:**

Porcine Model.

**Setting:**

Laboratory.

**Methods:**

Porcine tracheas were intubated with a 7.5 air‐filled polyvinyl endotracheal tube. A tracheostomy was performed. Monopolar and bipolar cautery were used in independent experiments to assess the ignition capacity. Seven trials were performed for each fraction of inspired oxygen (FiO_2_): 1.0, 0.9, 0.7, 0.6, 0.5, 0.4, and 0.3. The primary outcome was ignition of a fire. The time was started once the cautery function was turned on. The time was stopped when a flame was produced. Thirty seconds was used as the cut‐off for “no fire.”

**Results:**

The average time to ignition for monopolar cautery at FiO_2_ of 1.0, 0.9, 0.8, 0.7, and 0.6 was found to be 9.9, 6.6, 6.9, 9.6, and 8.4 s, respectively. FiO_2_ ≤ 0.5 did not produce a flame. No flame was created using the bipolar device. Dry tissue eschar shortened the time to ignition, whereas moisture in the tissue prolonged the time to ignition. However, these differences were not quantified.

**Conclusion:**

Dry tissue eschar, monopolar cautery, and FiO_2_ ≥ 0.6 are more likely to result in airway fires.

Airway fires are a considered a “never‐event” in the operating room (OR). Although rare, airway fires can cause devastating complications, such as smoke inhalation, tracheal stenosis, and death.^1^ Fires have been reported during airway surgical procedures such as tracheostomy and tonsillectomy.^2^ Guidelines recommend several strategies to decrease the likelihood of airway fires, including using the lowest possible fraction of inspired oxygen (FiO_2_) for inhalation, saline‐filled tube cuffs, and avoiding monopolar cautery.^3^ However, 200 to 600 airway fires per year still occur in the United States.^4^

The “fire triangle” describes the necessary components to ignite an airway fire. The triangle is composed of heat, oxidizer, and fuel. Oxygen is the most common oxidizer used in airway surgery.^2^ The heat source can be either monopolar cautery, bipolar cautery, or laser, and the fuel can be airway tissue or an endotracheal tube (ETT). The components of the triangle can be adjusted to increase or decrease the likelihood of fire in the airway; however, the threshold conditions for each component remain imperfectly understood.

Previous models using live animals, mannequins, and organic tissues have been used to simulate airway fires.^5^, ^6^, ^7^ However, live animal models are costly, and ethical considerations are the inherent limitations of these studies. Mannequin models allow for simulations that have structures closest to the human airway, but are not composed of organic tissue. Finally, organic tissue models use tissues derived from outside the airway.^8^ Given these limitations, the objective of this study was to design an organic airway tissue model to establish ideal conditions for starting an airway fire.

## Material and Methods

### Pig Larynx, Trachea, and Lungs

Pig airways (larynx, trachea, and lungs) were obtained from adult pigs (*Sus domesticus)* that were raised for human consumption. The experimental model consisted of a larynx, trachea and both lungs *en bloc*. Any residual muscle tissue was removed from the specimens surrounding the larynx. Western University Biosafety Committee approved the project. Animals were donated by a local meat processor in London, Ontario, Canada. The airways were maintained in a freezer at −40°C freezer and thawed to room temperature.

### Primary Outcome and Variables

Our primary outcome measure was the ignition of a fire while performing tracheostomy using electrocautery. Tracheostomy was selected as the procedure of choice because the surgical site is close to the oxygen source and it has been found to be the most common procedure resulting in airway fires.^2^ Different FiO_2_ levels were also examined_._ The two included energy source variables were monopolar and bipolar. The secondary outcome was the observation of any qualitative finding (such as eschar) that would make it more or less likely to ignite an airway fire.

### FiO_2_ Experiment

**Figure 1:**
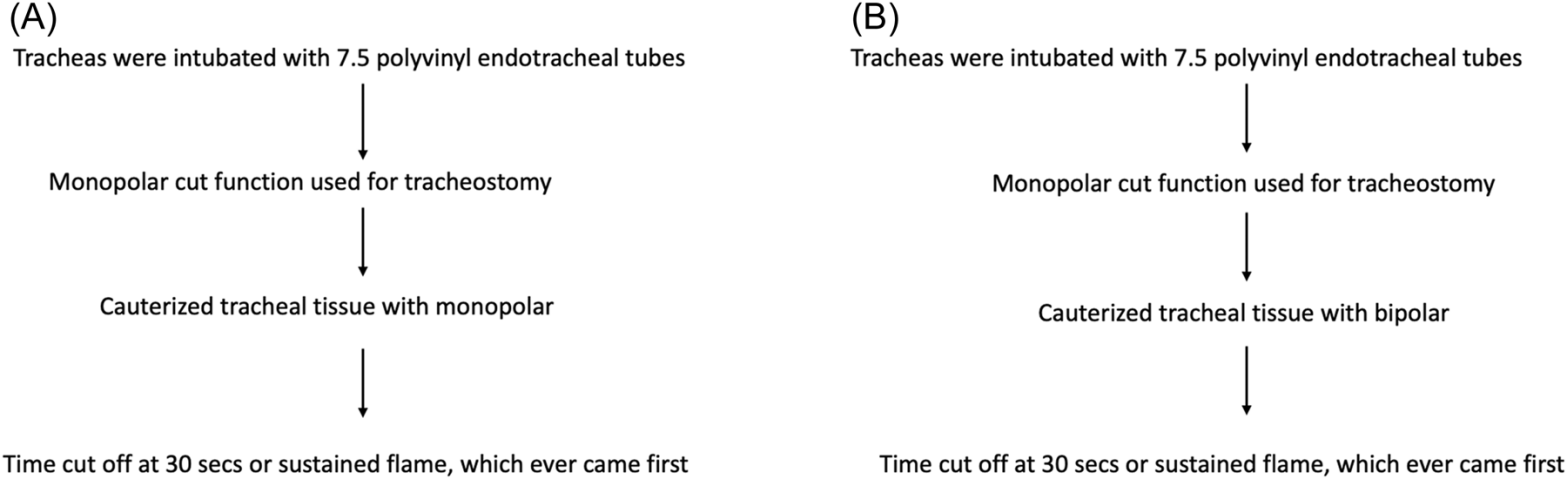
Flow chart of monopolar experiment (A) and bipolar experiment (B).

56 pig tracheas were intubated with a 7.5 polyvinyl ETT (Medtronic). Seven trials were completed for each FiO_2_: 1.0, 0.9, 0.7, 0.6, 0.5, 0.4, and 0.3. The flow rate was set at 15 L/min. FiO_2_ concentrations at the cauterization site were determined using an oxygen analyzer (CY‐12C). An electrocautery device (Bovie Aaron 2250™) was used to perform a tracheostomy using monopolar cautery, while bipolar cautery was used in independent experiments to assess the capacity to ignite a flame. In both the monopolar and bipolar trials, the airway was entered using the cut function of the monopolar. The ETTs were held in a position consistent with that in all trials. Monopolar trials began once the monopolar coagulation function was started. In bipolar trials, the time began when the bipolar was turned on. An arbitrary energy level of 40 W was chosen for monopolar cautery, and bipolar cautery was set at 40 W in all cases, as the energy level agreed upon by the authors. The time was stopped once a sustained flame was observed or 30 s was reached (Figure 1).

### Statistical Analysis

Fisher's exact test was used for statistical analysis of monopolar trials. All statistical analyses were performed using IBM SPSS Statistics version 25.0. Statistical significance was set at *p* ≤ .05.

## Results

### Optimizing Porcine Airway Model

**Figure 2:**
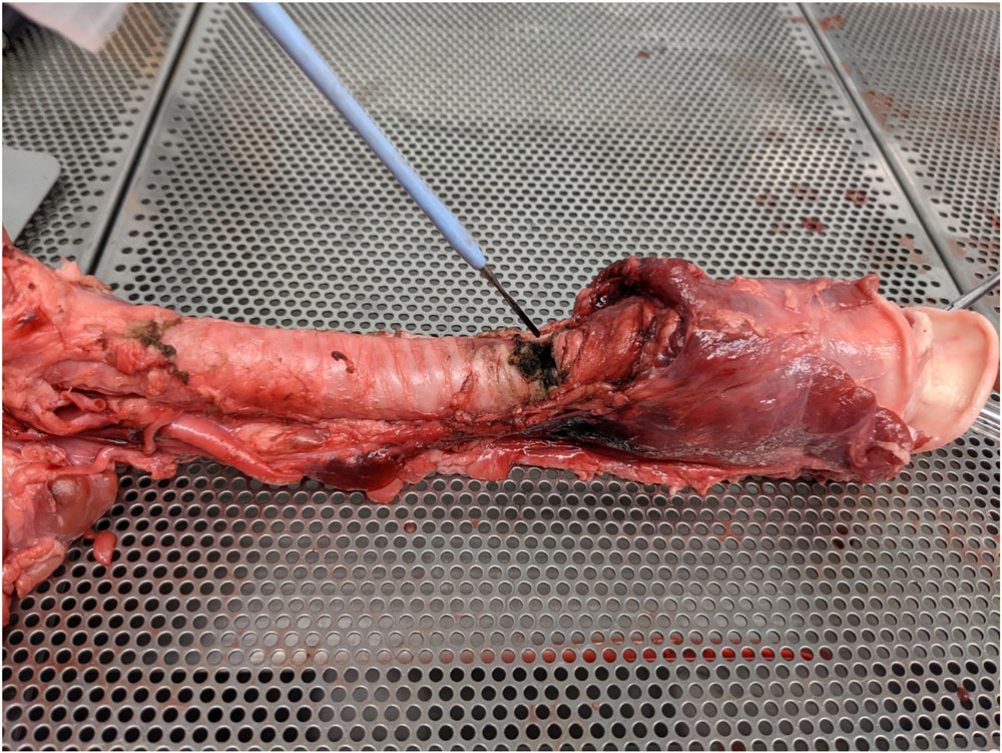
Eschar pig trachea intubated with and polyvinyl endotracheal tube.

To identify the ideal conditions for a consistent fire, FiO_2_ 1.0 at a flow rate of 15 L/min was used with monopolar cautery while performing tracheostomies. Several general trends are observed. First, it is difficult to garnish a flame when the tissue is wet. When the tissue began to build up eschar or char, flames were produced more easily (Figure 2). However, these observations have not been quantified.

### Varying FiO_2_ Concentrations

**Figure 3:**
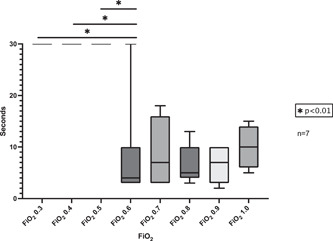
Creation of sustained flame from incremental FiO_2_. Once a sustained flame was observed time stopped. The cut off time was 30 s.

Seven trials were performed with incremental increases in the FiO_2_ concentrations (Figure 3). An FiO_2_ of 0.5 and under did not produce a flame with monopolar cautery. FiO_2_ of 0.6 and above, produced a flame. There were no statistical differences between the 0.6, 0.7, 0.8, 0.9, and 1.0. There was a statistically significant difference in the time to flame between 0.3 to 0.6, 0.4 to 0.6, and 0.5 to 0.6 (*p* = .005, *p* = .005, and *p* = .01, respectively).

### Bipolar Experiment

Using bipolar cautery, no flame could be ignited regardless of the FiO_2_ or trial duration, even in trials over two minutes. Seven trials were completed with a FiO_2_ of 100% with no flame generation.

## Discussion

The objective of this study is to create an organic airway‐specific model with the capability to create predictable and consistent fires and quantify the ideal conditions that result in ignition. It was found that (a) the pig trachea anatomy is similar to that of humans, as described previously,^9^ (b) dry tissue eschar was more likely to start a fire, (c) FiO_2_ of 0.6 or above was more likely to create an airway fire, and (d) using bipolar cautery does not result in airway fire even at FiO_2_ of 1.0.

Other models for the creation and simulation of airway fires have been previously described. A mannequin model ^7^ was used to test the effectiveness of a CO_2_ laser in creating airway fires. While this model was airway‐specific, the mannequin was made of a soft petroleum‐based inorganic material rather than an organic tissue, as in our model. Interestingly, the same group used raw chicken carcasses as an oropharyngeal model to create airway fires.^6^ Although this was a biological tissue, it was not airway specific. Live animal models have been used previously^5^, ^10^; however, there are many ethical issues and the number of replicates is limited owing to ethical concerns and costs. The model described in the present study has three main advantages: it is airway‐specific, relatively replicable, and uses organic tissues.

The initial phase of the study aimed to describe the conditions that are more likely to generate a reliable flame and utilize 100% FiO_2_ to achieve this goal. In general, dry eschar on tracheal tissue appeared to create more arcs from monopolar cautery and therefore more readily produced a flame, although the difference was not quantified. Moist or wet tissue appeared to decrease the likelihood of a flame.

The 2013 American Society of Anesthesiology guidelines recommend using the lowest allowable inspired oxygen, preferably less than 30%.^3^ However, prior to this study, it was unclear whether there was an FiO_2_ “threshold” that must be met to start a fire. Interestingly, Roy and Smith found in their oropharyngeal model that 0.5 FiO_2_ was the threshold to start a fire.^6^ However, the replicates were limited and the results did not reach statistical significance. Our study demonstrated that FiO2 less than or equal to 0.5 did not produce an airway fire. In addition, there was no significant difference in time to ignition between 0.6, 0.7, 0.8, 0.9, and 1.0 FiO_2_ concentrations in time to ignition. It seems that once a “threshold” of 0.5 FiO_2_ is crossed, the specific FiO_2_ level does not affect the time to which a sustained flame is created.

Other studies suggest that a threshold of 0.5 for the ignition of airway fire may be uncertain, despite the results in the present study. Ilgner et al^11^ reported a case of CO_2_ laser airway fire with an FiO_2_ of 0.3. Other cases of airway fire with FiO2 less than 0.5 have also been reported.^12^, ^13^ It is possible that FiO_2_ at the surgical site may be higher than the measured inspired and expired oxygen levels. Remz et al^14^ found that it took up to eight minutes for expiratory oxygen to reach 30% after dropping FiO2 to 0.3 if pre‐oxygenation was performed with 1.0 FiO_2_. Although end‐tidal control modes accelerate this process, oxygen content at the cauterization site remains unknown. This may explain the discrepancies in the literature.

In this study, bipolar cautery did not result in flame ignition at any FiO_2_. Within the literature, there is a single case report describing ignition of an airway fire using bipolar cautery in a tonsillectomy case.^15^ However, the anesthetic includes nitrous oxide, a well‐known oxidizer that supports airway fires, as well as a throat pack that might act as an additional fuel. Further studies are needed to confirm the superior safety profile of bipolar cautery for the prevention of airway fires.

This study had several limitations. Although pig airways are anatomically similar to human airways, they are not completely equivalent, and these differences may not allow the results to be completely transferrable to humans. We could not control for anatomical differences between the porcine airways, which could limit the applicability of these results. Live animal trials were not performed, further confounding the effects of variables such as body temperature and airway humidification, which could be present in vivo. Also, the tracheas were frozen for storage and were thawed for the experiments. This could introduce variability between trials if there was incomplete thawing that could skew the time of ignition. Additionally, most of the data was descriptive rather than quantitative, which might have introduced some bias into the study. These experiments were a proof of concept of our model that allows for consistent and reproducible fires. The next steps are to clarify other variables (cautery watts, humidity, etc.) that could alter the chances of igniting an airway fire. These experiments allows for future experiments to can be used in human airway models.

## Conclusion

An organic airway‐specific model was developed in this study, which allowed us to test ideal conditions for an airway fire. Dry tissue eschar is more likely to start a fire with a FiO_2_ of at least 0.6. Finally, bipolar cautery did not cause airway fires even with an FiO_2_ of 1.0. These data could help surgeons and anesthesiologists to understand the ideal conditions for generating an airway fire. Avoiding these conditions may help prevent devastating complications.

## Author Contributions

**Andrew M. Bysice**, data analysis, drafting, final approval, and accountability; **Tyler Oswald**, data analysis, drafting, final approval, and accountability; **Luis E. Mendoza Vasquez**, data analysis, drafting, final approval, and accountability; **Francisco Laxague**, data analysis, drafting, final approval, and accountability; **M. Elise Graham**, data analysis, drafting, final approval, and accountability; **Ruediger Noppens**, data analysis, drafting, final approval, and accountability; **Kevin Fung**, data analysis, drafting, final approval, and accountability.

## Disclosures

**Competing interests:** The authors declare no conflicts of interest.

## Funding source

None.
